# Comparison of Carlevale and Artisan retropupillary iris-claw intraocular lens fixation for managing aphakia: a systematic review and meta-analysis

**DOI:** 10.1007/s00417-026-07263-8

**Published:** 2026-05-06

**Authors:** Omar Alghaith, Tiago Nelson de Oliveira Rassi, Negin Sanadgol, Ehtesham Shamsher, Victor Barreiros Pungirum, Tran Bao Nghi, Fatemeh Khabazianzadeh, Maurício Maia

**Affiliations:** 1 Institute of Ophthalmology, University College London, London, UK; 2 Department of Ophthalmology, Federal University of São Paulo, São Paulo, Brazil; 3 John Radcliffe Hospital, Oxford, UK; 4 University of Buenos Aires, Buenos Aires, Argentina; 5 University of Debrecen, Debrecen, Hungary; 6 Eye Research Centre, Khatam-al-Anbia Hospital, Mashhad, Iran

**Keywords:** Intraocular lens, Carlevale, Retropupillary Iris-claw, Aphakia

## Abstract

**Purpose:**

Carlevale and retropupillary iris-claw Artisan intra-ocular lenses (IOLs) treat aphakia without capsular support, but their relative performance is uncertain.

**Methods:**

PubMed, Embase and the Cochrane Library were searched to March 2025. Primary outcomes: best-corrected visual acuity (BCVA), surgically induced astigmatism (SIA), mean absolute refractive error (MARE) and mean refractive error (MRE). Secondary outcomes: operating time and postoperative complications. Random-effects meta-analysis with I² and incision-type subgroups was performed.

**Results:**

Five studies comprising 631 eyes (229 Carlevale, 402 iris-claw) met inclusion criteria. Mean age was 70.1 ± 14.1 years, 61.18% were male; follow-up ranged from 1.3 to 11.5 months. BCVA did not differ between groups (− 0.01 logMAR; 95% CI − 0.13 − 0.11; *p* = 0.91; I²=43%). Carlevale reduced SIA (− 0.53D; 95% CI − 1.03 to − 0.04; *p* = 0.03; I²=73.7%), however, the benefit was confined to corneal-incision iris-claw comparators, not scleral-incision. MARE showed no overall difference, yet corneal-incision iris-claw cases were less predictable (MD-0.32; 95% Cl: -0.62-0.19, *p* = 0.30, I² =81.7%). Carlevale produced a myopic shift relative to iris-claw (− 0.66D; 95% CI − 0.87 to − 0.46; *p* < 0.01; I²=31.3%). Carlevale procedures were 11.9 min longer (95% CI 5.2–18.6; *p* < 0.01; I²=80.2%). Complication rates were comparable overall except for fewer IOL dislocations with Carlevale (OR 0.16; 95% CI 0.03–0.87; *p* = 0.034; I²=0%).

**Conclusions:**

Both lenses provide similar visual acuity and safety in aphakic eyes lacking capsular support. Carlevale confers lower dislocation risk and greater refractive predictability relative to corneal-incision iris-claw implantation, at the expense of a longer operating time. Incision-related heterogeneity highlights the need for standardised surgical and reporting frameworks.

**Graphical Abstract:**

**Figure:**
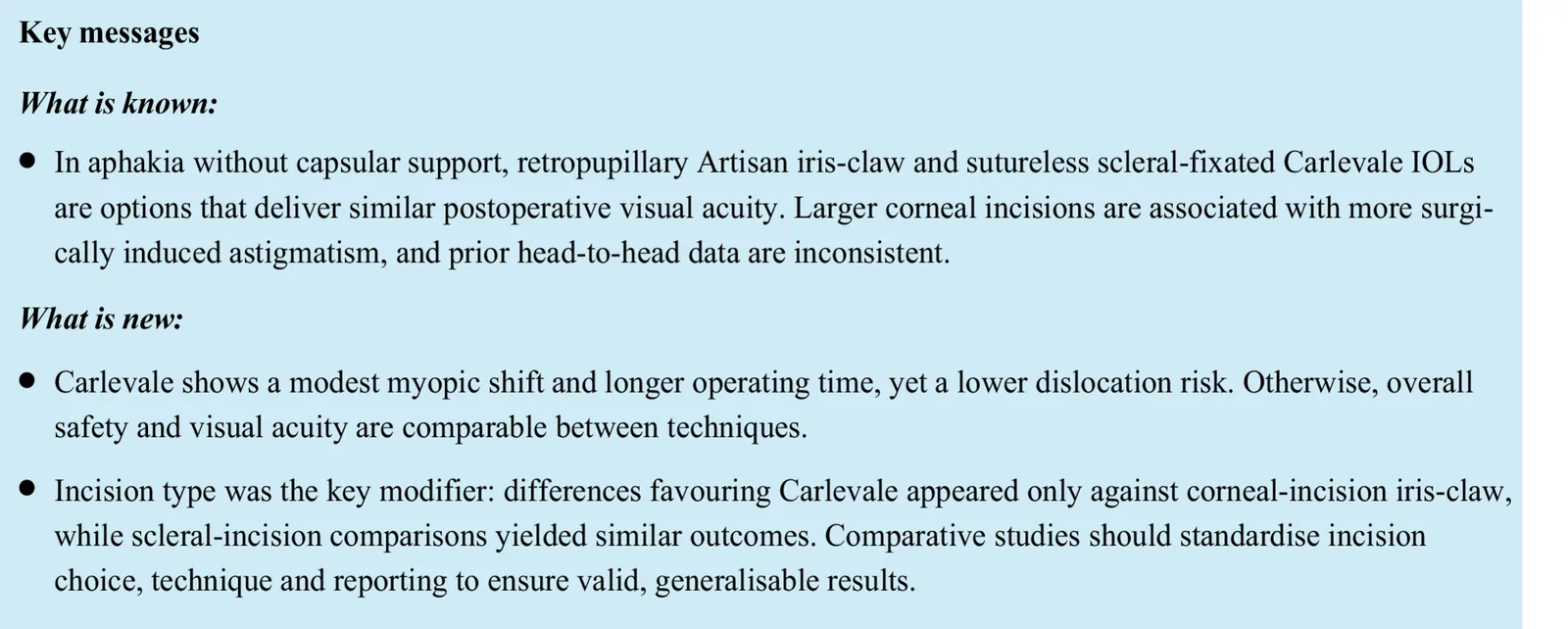

**Supplementary Information:**

The online version contains supplementary material available at https://doi.org/10.1007/s00417-026-07263-8.

## Introduction

Intraocular lens (IOL) implantation in the absence of capsular support remains a surgical challenge, with no consensus on the optimal approach [1]. Anterior chamber IOLs (ACIOLs), once favoured for their technical simplicity, have been abandoned due to the risk of corneal endothelial loss, glaucoma, and uveitis [2]. Scleral-fixated posterior chamber IOLs (SFIOLs) offer a widely used alternative, utilizing either transscleral sutures or sutureless techniques to secure the lens. However, sutured approaches are prone to long-term complications, with suture degradation leading to IOL dislocation in up to 27.9% of cases [3]. These limitations can be addressed with sutureless intrascleral fixation techniques [4, 5]. However, these methods often involve a steep learning curve and technical complexity, which may limit clinical applicability.

The iris-claw IOL has gained popularity for secondary implantation in aphakic eyes due to its relative technical simplicity and favourable anatomical and visual outcomes. Fixated to the mid-peripheral iris via enclavation, the lens can be placed retropupillary, offering stable posterior chamber positioning without the need for scleral sutures. Clinical studies have reported significant improvements in best-corrected visual acuity (BCVA) and refractive stability following implantation [6]. However, the Artisan lens is rigid and requires a large corneal or sclerocorneal incision (5–6 mm), which is associated with greater surgically induced astigmatism (SIA) [7]. Also, its dependence on healthy iris tissue limits use in cases of iris trauma or atrophy. Iris-related complications, including pigment dispersion, pupil ovalization, and chronic inflammation, have also been documented [1]. These limitations have prompted interest in alternative fixation strategies that avoid iris manipulation and minimize astigmatic shifts.

The Carlevale IOL (Soleko IOL Division, Pontecorvo, Italy) represents a novel sutureless scleral-fixated approach. It is a single-piece, foldable acrylic lens with two T-shaped haptics designed to anchor within scleral tunnels created approximately 1.5–2 mm posterior to the limbus [4, 8]. This plug-in fixation allows for stable posterior chamber implantation without the use of sutures or iris support. Unlike rigid PMMA lenses, the Carlevale can be inserted through a small, self-sealing incision (2.2–2.7 mm), reducing the risk of SIA [9–11]. By avoiding iris contact, the Carlevale technique minimizes iris-related complications and broadens its applicability to cases with poor iris integrity [4]. Early clinical series have demonstrated encouraging outcomes, with low rates of dislocation and favourable refractive profiles [8].

Comparative studies evaluating the iris-claw and Carlevale IOLs have yielded heterogeneous findings. While some studies found better refractive outcomes for Carlevale’s technique [11], others found Iris-claw presenting less refractive error and induced astigmatism [12]. Also, results concerning surgical time are conflicting. While some studies found the Iris-claw technique more time-efficient [13], others found no difference regarding the extent of surgery [9]. Safety profiles appear similar between groups, although variation in surgical approach may act as a confounding factor.

Considering these uncertainties, we performed a systematic review and meta-analysis to compare visual and refractive outcomes of the Carlevale and Artisan IOLs in aphakic eyes lacking capsular support. Additionally, we assessed the safety profiles of both techniques, aiming to support clinical decision-making regarding aphakia.

## Methods

We performed a systematic review and meta-analysis following the Cochrane Collaboration Handbook for systematic review of interventions and the preferred reporting items for systematic reviews and meta-analysis (PRISMA) statements [14, 15].

### Eligibility criteria

Inclusion in this meta-analysis was restricted to studies that met all the following eligibility criteria: (1) randomized trials or nonrandomized studies; (2) comparing Carlevale with retropupillary iris-claw fixation of Artisan; (3) enrolling Aphakic patients. In addition, studies were included only if they reported any of the clinical outcomes of interest. We excluded (1) abstracts; (2) case reports and (3) studies without intervention of interest.

### Search strategy and data extraction

We systematically searched PubMed, Embase, and the Cochrane Central Register of Controlled Trials. The search strategy is outlined in the Supplementary Appendix S1. References from included studies, previous systematic reviews, and meta-analyses were also manually searched for any additional studies. Two authors (O.A. and E.S.) independently extracted the data following predefined search criteria and quality assessment. The prospective meta-analysis protocol was registered on PROSPERO on January 21, 2025, under protocol CRD42025634728.

### Endpoints

Outcomes included post-operative BCVA, post-operative astigmatism, surgically induced astigmatism, mean operating time, mean refractive error (MRE), and mean absolute refractive error (MARE). Post-operative astigmatism was defined as total astigmatism after the intervention. Surgically induced astigmatism was defined as the difference between post-operative and pre-operative astigmatism. We defined MRE as the arithmetic difference between the achieved postoperative refraction and the preoperative target refraction, preserving the sign to indicate direction: positive values denoting a hyperopic shift and negative values a myopic shift. This metric reflects both the magnitude and directional tendency of deviation from the intended refractive outcome. We defined MARE as the absolute value of this difference, representing the overall deviation from target refraction regardless of direction.

### Quality assessment

Two independent authors completed the risk of bias assessment using ROBINS-I (V.P. and T.N.). (Sterne et al., 2016) Disagreements were resolved by consensus after the reasons for the discrepancy were discussed.

### Statistical analysis

Continuous outcomes were compared with mean differences. Odd ratios (OR) with 95% confidence intervals were used to compare treatment effects for categorical endpoints. We assessed heterogeneity with I^2^ statistics and Cochrane Q test; *p−values <* 0.10 and *I*^2^ *>* 25% were considered significant for heterogeneity, in line with Cochrane guidance.

In cases where the outcome of interest was not reported, values were imputed using standardized formulas in accordance with the Cochrane Handbook for Systematic Reviews of Interventions, which outlines methods for calculating change from baseline in the presence of missing data [15]. The results remained consistent and are outlined alongside the methods used in Supplementary appendix S2.

We used the DerSimonian and Laird random-effects model. We also performed a sensitivity analysis by removing each individual study from the outcome of assessment. We also performed subgroup analysis for the type of incision in the Iris-claw technique (cornel, corneal-scleral or mixed). We used RStudio version 4.4.2 for statistical analysis.

## Results

### Study selection and characteristics

**Fig. 1:**
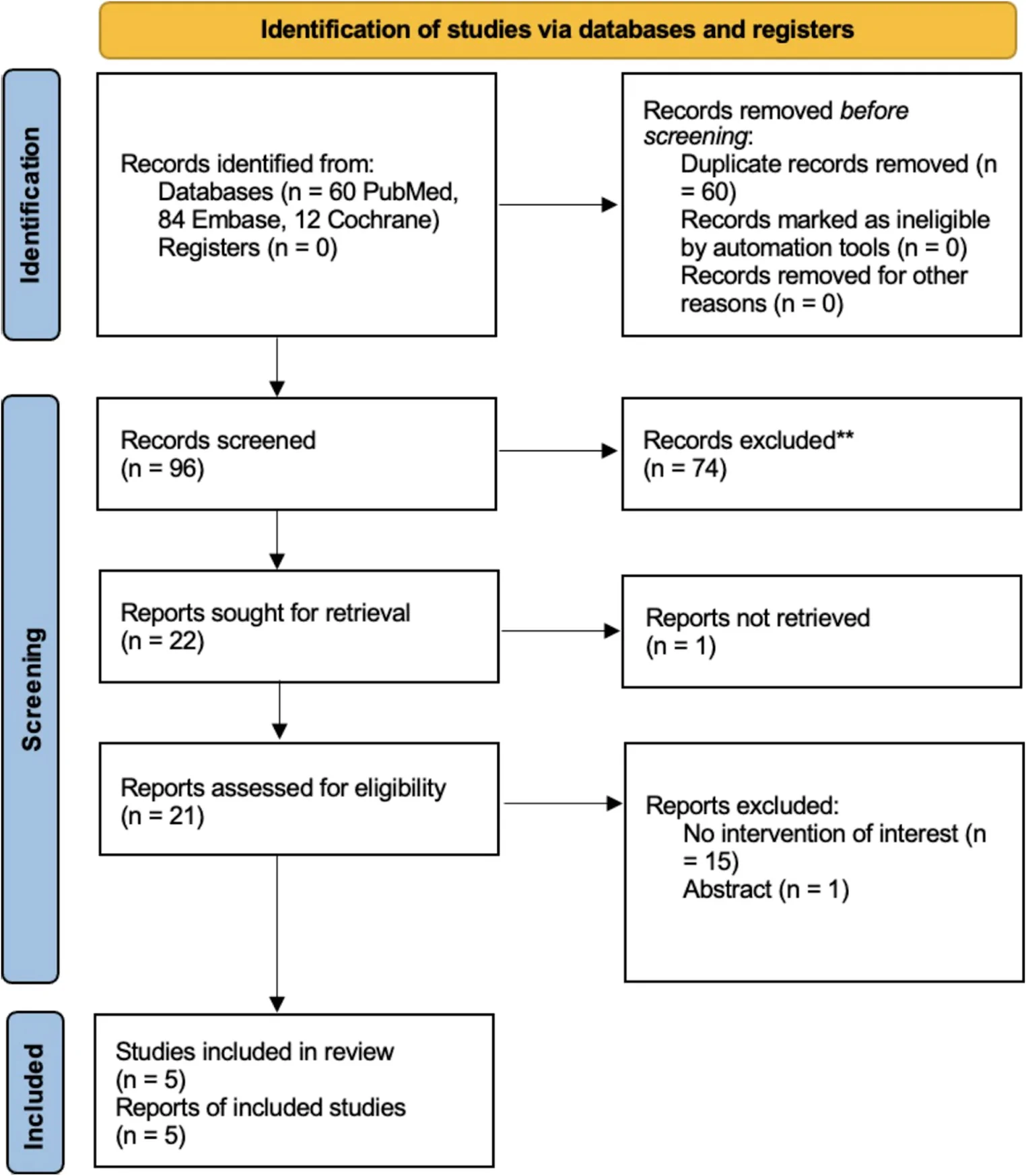
PRISMA flow diagram of the study selection process

The initial search yielded 156 results. After removal of duplicate records and ineligible studies, 21 remained and were fully reviewed based on inclusion criteria. Of these, a total of 5 studies were included, comprising 631 eyes (Fig. 1). Figure S1 presents the flowchart of the study selection process. A total of 229 (36.3%) eyes received Carlevale IOL and 402 (63.7%) received Artisan IOL. Study characteristics are reported in Table 1 (Supplementary Appendix S3).

### Pooled analysis of all studies

**Fig. 2:**
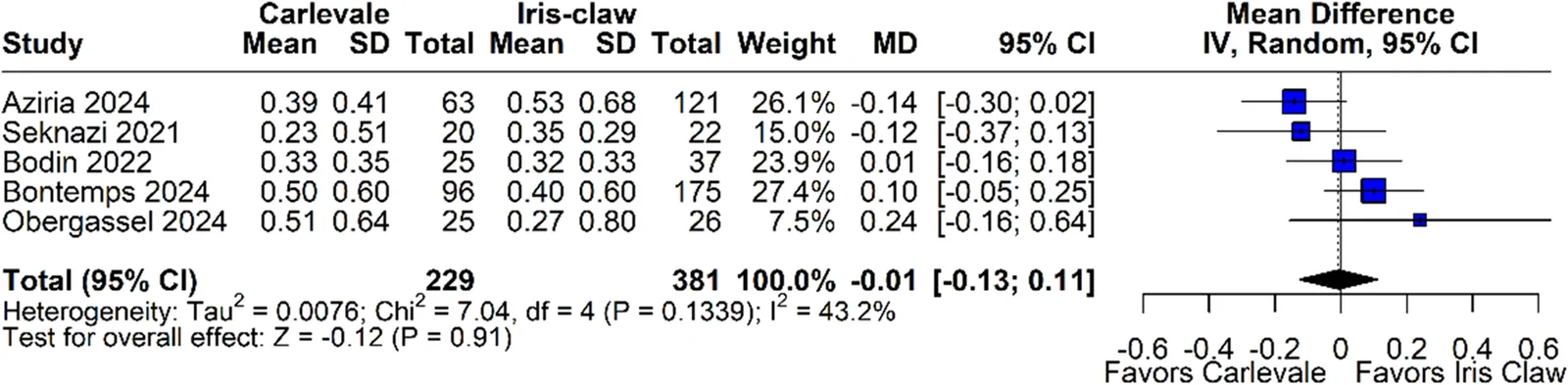
No difference in post-operative BCVA between Carlevale and Iris-claw groups (MD -0.01 logMAR; 95% Cl: -0.13 to 0.11; *p* = 0.91; I2 = 43.2%)

There was no statistically significant difference between groups in post-operative BCVA (MD -0.01 logMAR; 95% Cl: -0.13 to 0.11; *p* = 0.91; I^2^ = 43.2%, Fig. 2). Leave-one-out sensitivity analysis showed Aziria et al. as an influential outlier, as its removal led to a considerable decrease in heterogeneity, however without changing the overall effect.

**Fig. 3:**
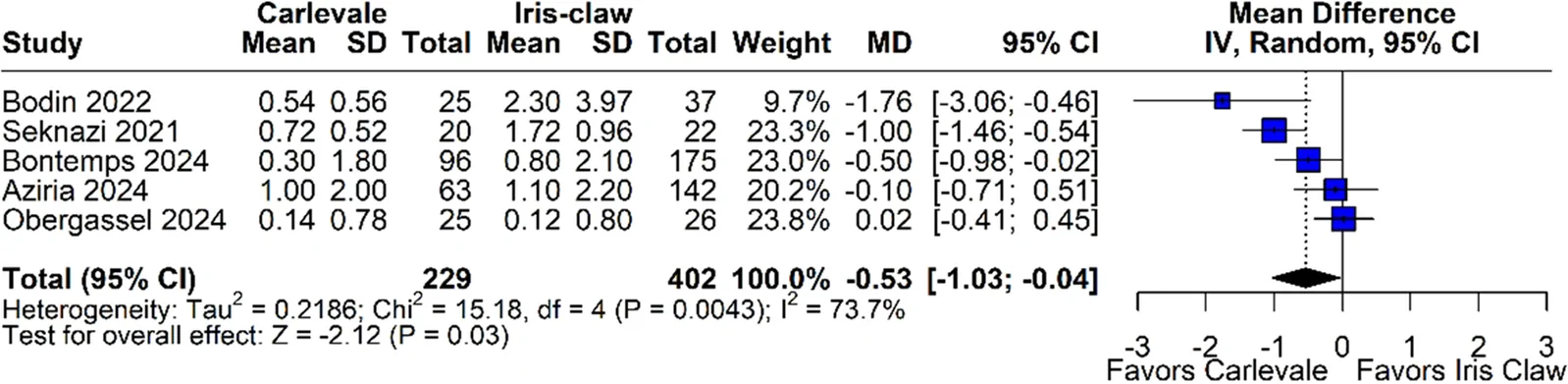
Surgically induced astigmatism (SIA) favoured Carlevale group, with high heterogeneity (MD -0.53D; 95% CI: -1.03 to -0.04; *p* = 0.03; I2 = 73.7%)

**Fig. 4:**
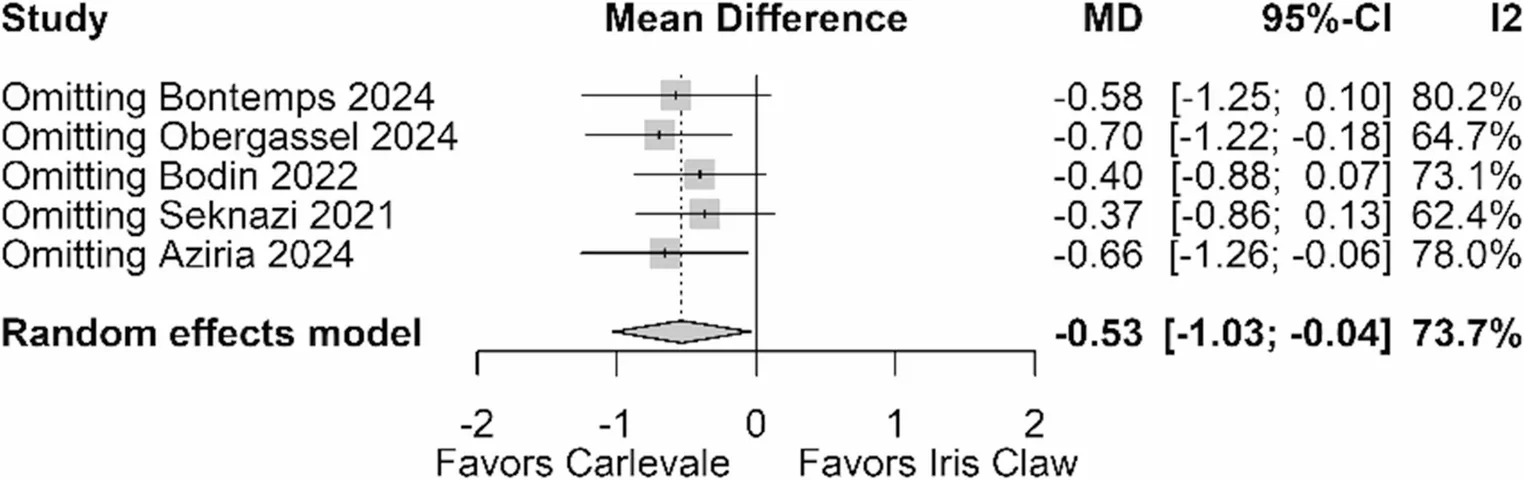
Leave-one-out sensitivity analysis for surgically induced astigmatism (SIA). Omitting individual studies did not substantially reduce heterogeneity (I² remained between 62.4% and 80.2%), indicating that no single study accounted for the overall inconsistency

In patients receiving Carlevale IOL, there was a trend toward less surgically induced astigmatism (MD -0.53D; 95% CI: -1.03 to -0.04; *p* = 0.03; I^2^ = 73.7%, Fig. 3), however, with high inconsistency. Our leave-one-out sensitivity analysis did not identify an individual study responsible for the high heterogeneity (Fig. 4).

**Fig. 5:**
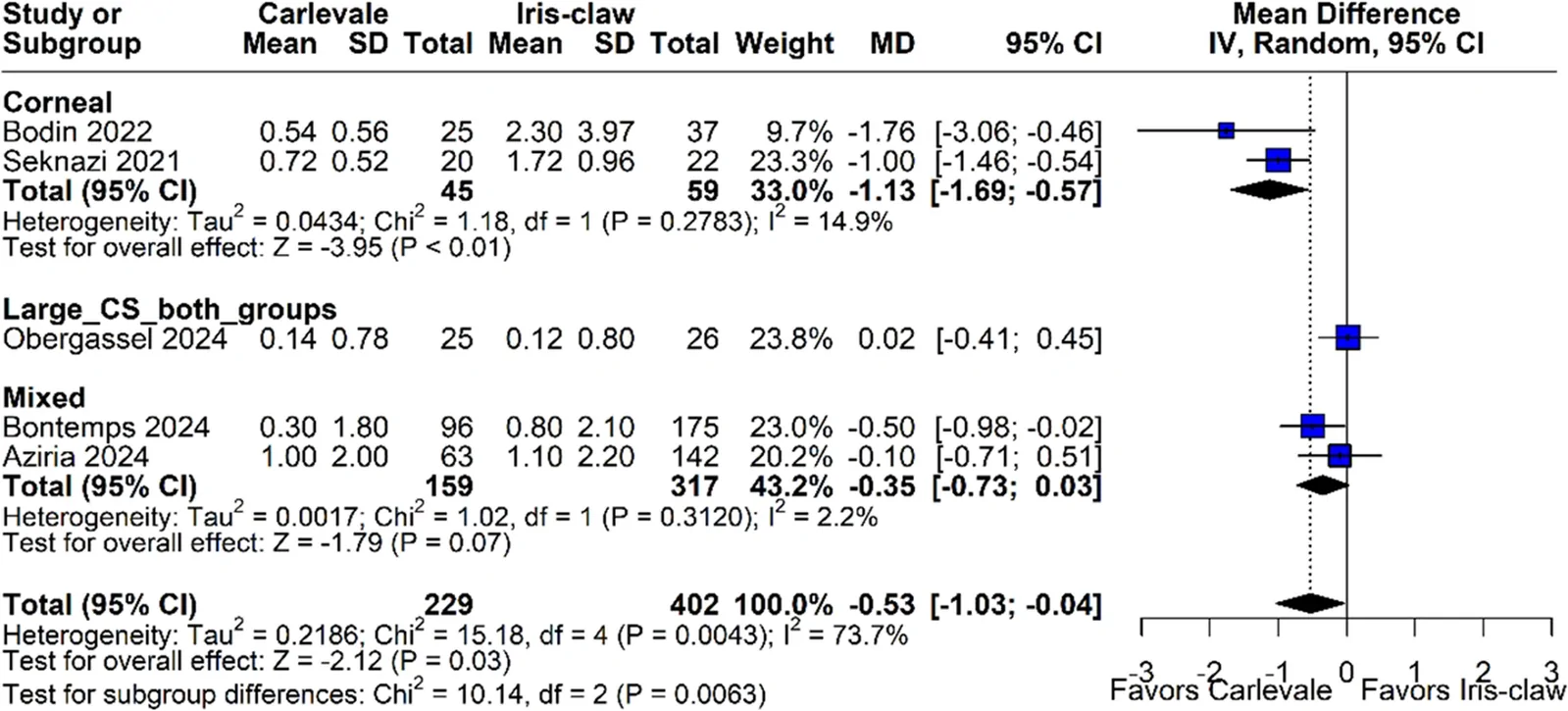
Subgroup analysis of surgically induced astigmatism (SIA) stratified by incision type used for retropupillary iris-claw implantation

However, in our subgroup analysis, we found that the type of incision used in the iris claw technique was the primary source of this inconsistency. As we separated these groups, the heterogeneity dropped considerably. In the mixed sclerocorneal incision subgroup, there was no statistically significant difference in SIA between Carlevale and iris-claw IOLs (MD − 0.35 D; 95% CI: − 0.73 to 0.03; *p* = 0.07; I^2^ = 2.2%, Fig. 5). However, in the corneal incision subgroup, the Carlevale group demonstrated significantly lower SIA compared to iris-claw IOLs (MD − 1.13 D; 95% CI: − 1.69 to − 0.57; *p* < 0.01; I^2^ = 14.9%, Fig. 5)

**Fig. 6:**
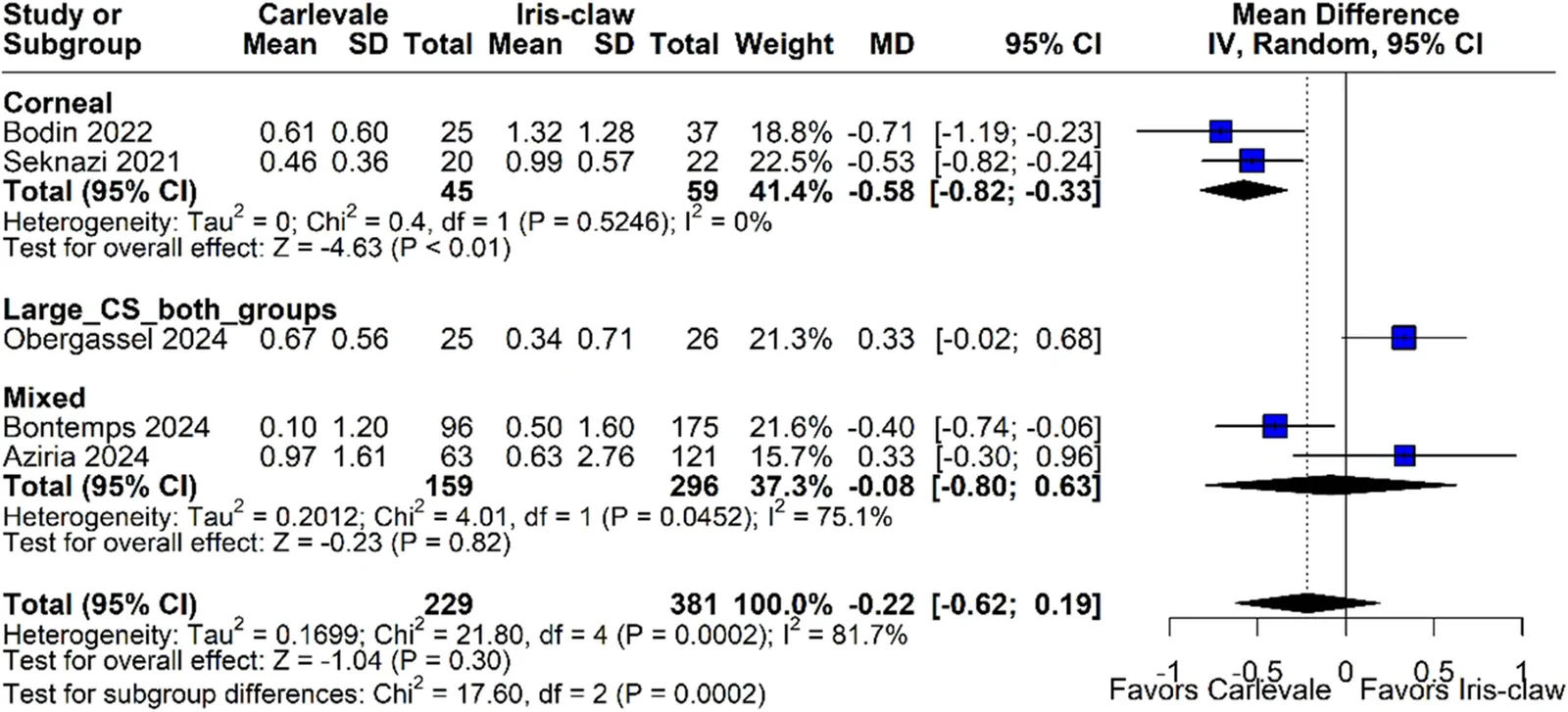
Forest plot comparing postoperative astigmatism between Carlevale and retropupillary iris-claw intraocular lenses. No statistically significant difference was observed between groups, with no heterogeneity detected (I^2^ = 0%)

**Fig. 7:**
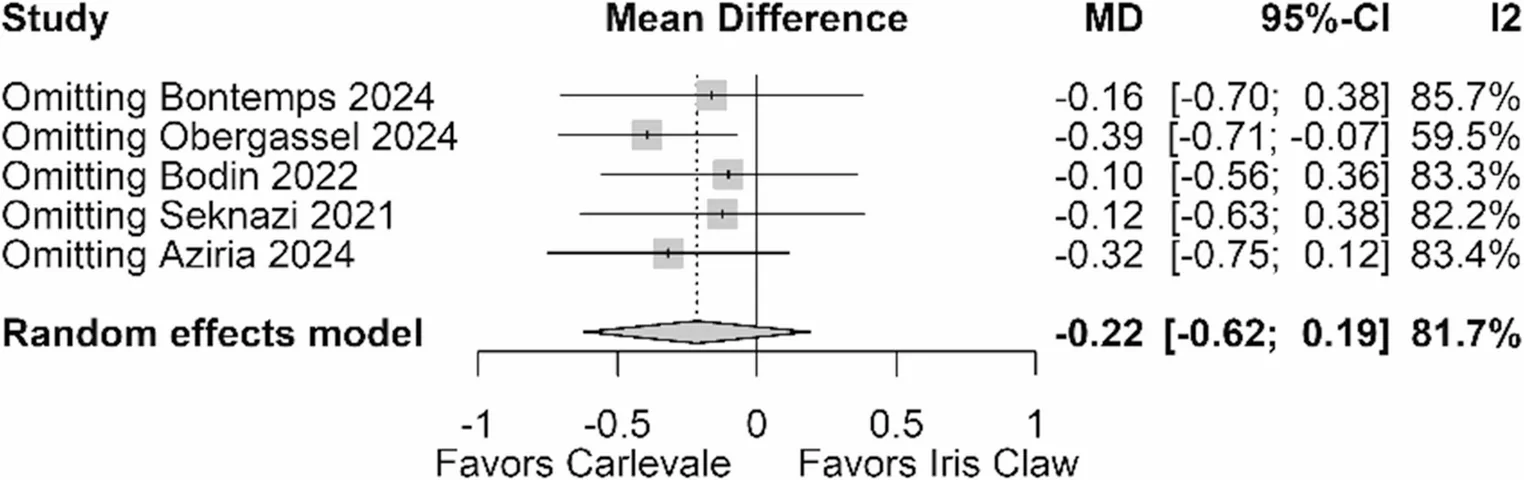
Forest plot of mean absolute refractive error (MARE) comparing Carlevale and retropupillary iris-claw intraocular lenses. No overalldifference was observed between groups, with substantial heterogeneity across studies

**Fig. 8:**
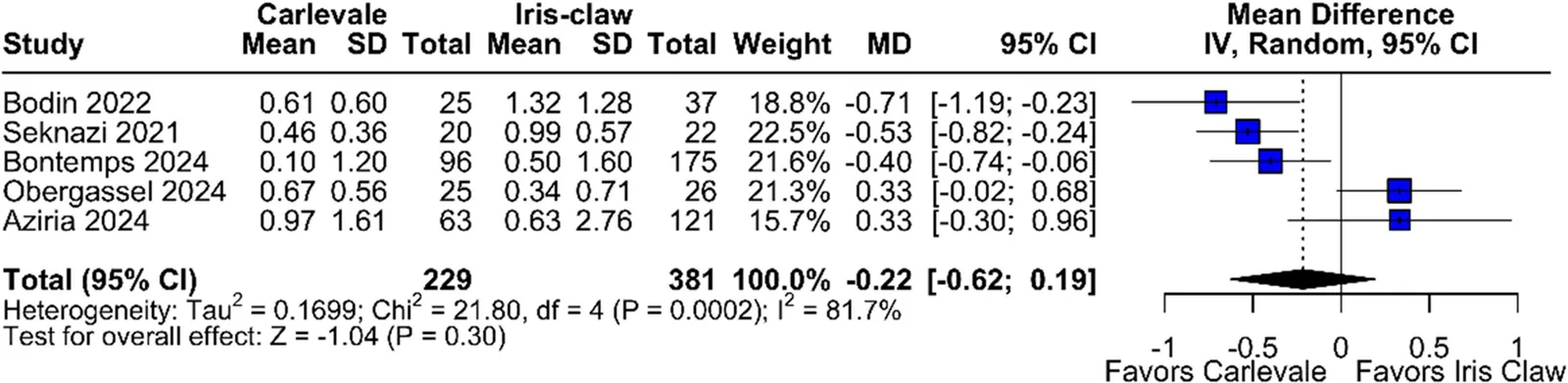
Leave-one-out sensitivity analysis for mean absolute refractive error (MARE). Exclusion of individual studies did not substantially reduce heterogeneity (I^2^ range: 59.5% to 85.7%), indicating no single study accounted for the observed inconsistency

**Fig. 9:**
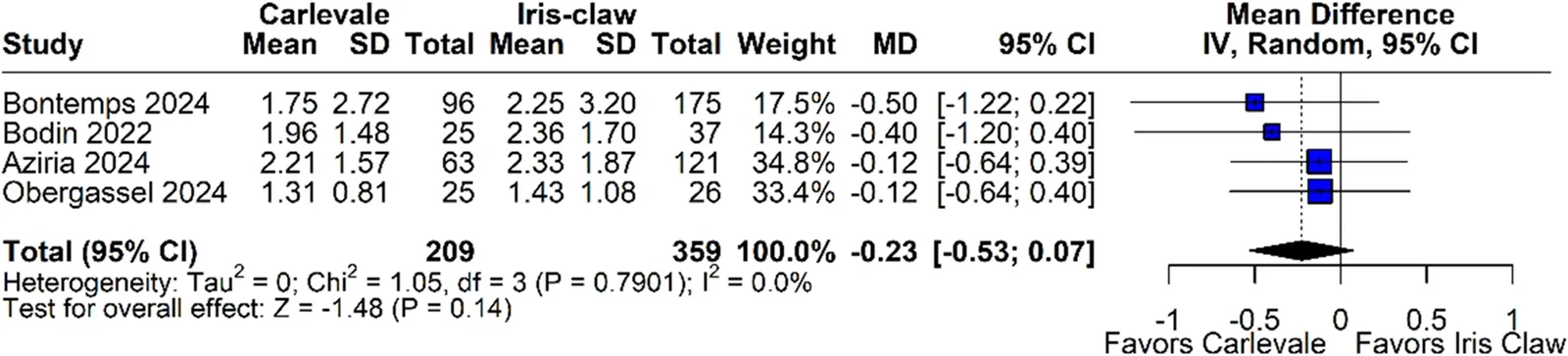
Subgroup analysis of mean absolute refractive error (MARE) stratified by incision type. The corneal incision subgroup demonstrated significantly more predictable refractive outcomes favouring Carlevale intraocular lenses, with resolution of heterogeneity

There was no statistically significant difference between groups in post-operative astigmatism (MD -0.23D; 95% Cl: -0.53 to 0.07; *p* = 0.14; I^2^ = 0%, Fig. 6). Concerning MARE, we found no differences between groups; however, there was significant heterogeneity (MD -0.32; 95% Cl: -0.62 to 0.19, *p* = 0.30, I^2^ = 81.7%, Fig. 7). Leave-one-out analysis could not identify an individual study responsible for the high heterogeneity (Fig. 8); however, our subgroup analysis found that, again, the variation in incision type of the iris claw technique was accountable for the high heterogeneity. The corneal incision subgroup presented with significant favourable results towards Carlevale’s technique, with more predictable refractive outcomes (MD -0.58; 95% Cl: -0.82 to -0.33, *p* < 0.01, I^2^ = 0%, Fig. 9).

**Fig. 10:**
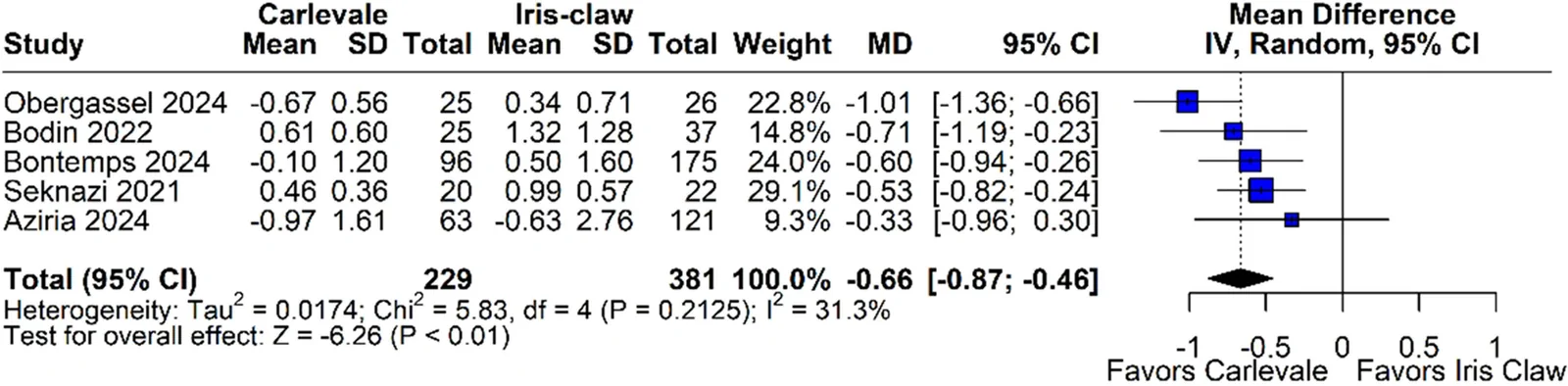
Forest plot comparing mean refractive error (MRE) between Carlevale and retropupillary iris-claw intraocular lenses. Carlevale intraocular lenses were associated with a significantly more myopic refractive outcome (MD -0.66 D, 95% Cl -0.87 to -0.46; *p* < 0.01)

**Fig. 11:**
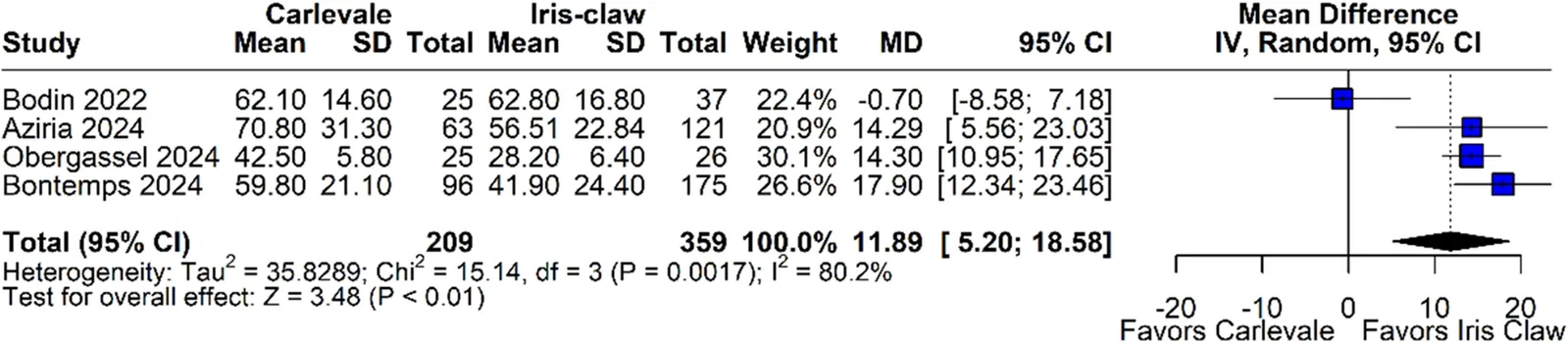
Forest plot comparing mean operating time between Carlevale and retropupillary iris-claw intraocular lens implantation. Carlevale procedures were associated with significantly longer operating times, with substantial heterogeneity across studies

**Fig. 12:**
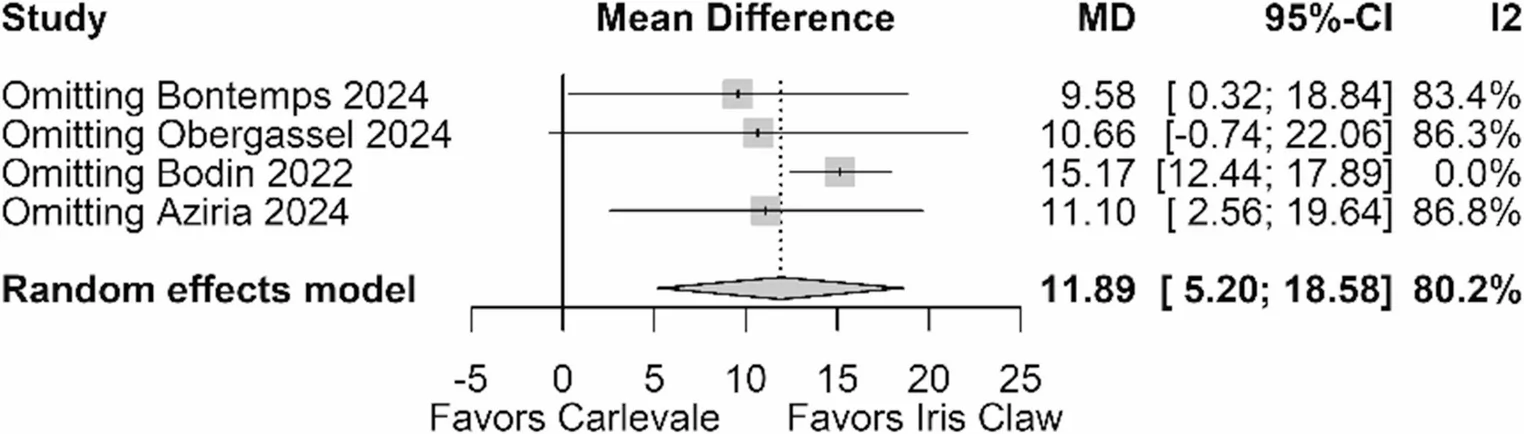
Leave-one-out sensitivity analysis for mean operating time. Exclusion of Bodin et. al eliminated heterogeneity (I^2^ = 0%) and increased the pooled estimate, identifying this study as the primary contributor to variability

Carlevale IOLs were associated with a more myopic refractive outcome (MD -0.66D; 95% CI: -0.87 to -0.46; *p <* 0.01; I^2^ = 31.3%, Fig. 10), and longer mean operating time (MD 11.89 min; 95% CI: 5.20 to 18.58; *p* < 0.01; I^2^ = 80.2%, Fig. 11). Leave-one-out analysis of mean operating time identified Bodin et al. as an outlier, upon its removal, heterogeneity dropped to I^2^ = 0%, and the refined pooled estimate increased to 15.17 min (95% CI: 12.44 to 17.89; *p* < 0.01, Fig. 12).

We analysed the following complications: IOL dislocation, cystoid macular oedema, hypotony, retinal detachment, intravitreous haemorrhage, hyphaema and found no difference between Carlevale and iris-claw, except for IOL dislocation. Carlevale demonstrated a significant reduction in odds of IOL dislocation compared to iris-claw (OR 0.16; 95% CI; 0.03 to 0.87; *p* = 0.034, I^2^ = 0%, Fig. S5.1).

### Quality Assessment

The ROBINS-I tool was used for quality assessment [16]. One study was considered at high risk of bias (Fig. S6). The strength of evidence was assessed using the GRADE framework (Fig. S11) [17].

In funnel plot analysis, studies occupied a symmetrical distribution according to weight and converged toward the pooled effect as weight increased. Egger’s test indicates no evidence of publication bias *p* = 0.61; Fig. S7).

## Discussion

In this systematic review and meta-analysis of 5 studies and 631 eyes, we compared Carlevale IOL to retropupillary iris-claw Artisan IOL in patients with poor capsular support. The main findings with Carlevale IOL implantation include: (1) Comparable BCVA outcomes to iris-claw; (2) Less SIA and more predictable refractive outcomes when compared to corneal incision iris-claw technique; (3) trend of myopic shift; and (4) longer mean operating time. Also, the safety profile seems similar between both techniques, but with a considerably higher risk of IOL dislocation when using the iris claw technique. The efficacy and safety of this technique could not be compared to the iris claw corneoscleral technique due to insufficient data.

Postoperative best-corrected visual acuity (BCVA) is a primary concern following ophthalmic surgery. In our analysis, both Carlevale and iris-claw intraocular lens (IOL) groups demonstrated improvement in visual acuity, with no difference between the two techniques. These findings align with previous literature indicating that, despite differences in surgical techniques and fixation methods, both Carlevale and iris claw IOLs yield comparable visual outcomes in aphakic patients [18, 19]. Broader comparisons across various fixation techniques, including anterior chamber iris-claw, Sutureless intrascleral, and Carlevale IOLs have similarly demonstrated no significant differences in postoperative BCVA outcomes [20].

Surgically induced astigmatism (SIA) is primarily influenced by the size and location of the surgical incision, as well as the presence or absence of sutures [7]. The sutureless Carlevale in intraocular lens (IOL), which requires a smaller 2.2 mm corneal incision, is expected to induce less SIA than the Artisan iris-claw IOL, which necessitates a larger 5–6 mm incision. In our analysis, SIA demonstrated a trend toward lower values in eyes receiving Carlevale IOLs. However, high heterogeneity persisted (I^2^ = 81.7%). We found in our subgroup analysis that the source of this inconsistency was the type of the incision used in the iris-Claw technique. Corneal incisions appeared to have the worst refractive results for this technique; This is further supported by Aziria et al., who found that the corneoscleral incision Iris Claw group had similar postoperative astigmatism and SIA compared to the Carlevale group, while the corneal incision group showed the worst refractive outcomes among the three. This was also demonstrated in a subgroup analysis for SIA by Bodin et al. where they found no difference between Carlevale’s and scleral iris claw technique for SIA.

The high inconsistency in the overall MARE was also easily explained by this hypothesis, with a clear benefit towards the Carlevale’s technique when the iris claw technique was performed with a corneal incision (as shown by our subgroup analysis). Strong evidence supports that the use of scleral incisions is superior in terms of refractive outcome [21]. Concerning post-operative astigmatism, our analysis found it to be comparable between groups, consistent with the findings of Van Severen et al. [18]. However, we question the validity of this outcome for measuring post-surgery refractive efficacy. Differently from SIA, postoperative astigmatism reflects the total astigmatic value after surgery and does not distinguish between pre‑existing and newly induced components.

Our analysis demonstrated a significant myopic shift in patients undergoing Carlevale IOL implantation compared to those receiving retropupillary iris-claw IOLs. This difference may be attributed to variations in incision type and/or in haptic fixation depth and scleral tunnel size, both of which influence effective lens position (ELP). Given that Carlevale IOL haptics are fixated 1.5–2.5 mm posterior to the limbus, variability in haptic positioning and externalization depth may result in anterior optic displacement, increasing refractive power and inducing a myopic shift relative to the more anterior and stable fixation plane of retropupillary Artisan lenses. This is consistent with previous findings reporting a greater myopic deviation in eyes implanted with Carlevale IOLs (0.46 ± 1.35 D) compared to those with Artisan IOLs (0.08 ± 1.60 D, *p* = 0.019) [18]. This proposed mechanism is further supported by AS-OCT data comparing Carlevale and Yamane IOLs demonstrated a trend toward greater myopic prediction error with Carlevale and revealed considerable variation in effective lens position within the Carlevale group, with a strong positive correlation between ELP and prediction error [22].

Surgical duration is an essential factor in IOL selection, particularly in eyes with compromised capsular support. One previous study has reported that iris-claw IOL implantation is associated with shorter operating times compared to sutureless scleral-fixated IOLs (SSF IOLs), such as the Carlevale lens [18]. This trend was mirrored in our analysis (Fig. 6). In the included studies [9, 10, 12, 13], implantation of the Carlevale IOL involved the creation of two scleral flaps positioned 180° apart and the use of 25-gauge sclerotomies beneath each flap to externalize the T-shaped haptics. The plugs were then secured beneath the scleral flaps. While incision size and the use of pars plana vitrectomy varied across studies, the core fixation steps remained consistent. Retropupillary Artisan IOL implantation in the included studies involved a 5.5 to 6 mm corneal or sclerocorneal tunnel incision centred at the 12 o’clock position. The lens was inserted through the main incision and positioned behind the iris. Enclavation of the haptics to the mid-peripheral iris was performed through one or more paracenteses. Specific paracentesis positions were not consistently reported. The main incision was closed with sutures in all cases. A leave-one-out sensitivity analysis identified Bodin et al. [9] as the source of heterogeneity (I² = 80%). In this study, patients receiving iris-claw IOLs also underwent a standardized 3-port, 25-gauge pars plana vitrectomy. This increased surgical time may explain the lack of time difference between Carlevale and Artisan in their dataset. Exclusion of Bodin et al. eliminated the observed heterogeneity and produced a pooled effect estimate consistent with the trend observed across the remaining studies.

We compared the safety profiles of both IOLs. No significant differences were found in overall safety between the two techniques. However, an important trend toward lower IOL dislocation rates with Carlevale was observed. This may be explained by Carlevale’s T-shaped haptic anchors, which provide stable intrascleral fixation and reduce the risk of tilt or decentration [12], as well as its independence from iris tissue, thereby avoiding the instability seen with iris-claw designs. The higher dislocation rate associated with iris-claw IOLs may stem from their reliance on iris tissue for fixation. In a recent study by Choi et al., 9.7% of 103 eyes that underwent retropupillary iris-claw IOL implantation experienced disenclavation over two years, with significantly more events in eyes with iris atrophy. The authors note that surgical difficulty in visualizing the posterior iris may lead to insufficient enclavation, suggesting that both anatomical and technical limitations contribute to iris-claw instability. These findings highlight a key limitation of retropupillary iris-claw fixation and support the greater stability observed with scleral-fixated designs like Carlevale.

Our study has important limitations. First, the meta-analysis included a limited number of studies, comprising a total of 631 eyes, which restricts the generalisability of our findings. Second, none of the included studies were randomised, which increases susceptibility to selection bias and limits causal inference. To mitigate this, we employed random-effects models, conducted sensitivity and subgroup analyses, and assessed risk of bias using the ROBINS-I tool, alongside certainty of evidence using the GRADE framework. Third, there was an unequal distribution of intraocular lens types, with a greater proportion of eyes receiving Artisan iris-claw lenses. Although inverse-variance weighting within a random-effects framework was applied and sensitivity analyses did not materially alter the pooled estimates, this imbalance may still influence the robustness of comparative conclusions. Fourth, we were unable to evaluate IOL opacification in the Carlevale group. The hydrophilic lens material used in Carlevale IOLs carries a theoretical risk of opacification, particularly following vitrectomy or intraocular gas tamponade. However, these changes typically occur over extended follow-up periods, which were beyond the scope of the included studies. Lastly, a key methodological flaw in the existing literature is comparing outcomes based on different surgical incisions, specifically corneal versus sclerocorneal approaches. These inconsistencies contribute to outcome variability unrelated to IOL type. Superior refractive outcomes favouring Carlevale may be biased by variations in incision types used. Future comparative studies must standardize incision techniques to ensure unbiased results.

## Conclusion

This meta-analysis provides a comparative analysis of Carlevale and retropupillary iris-claw IOLs for the management of aphakia in eyes with poor capsular support. While both IOLs demonstrated comparable safety profiles, Carlevale IOLs showed a trend toward lower dislocation rates. Also, Carlevale IOLs were associated with more predictable refractive outcomes in comparisons involving corneal-incision iris-claw implantation. Comparison of this technique with scleral incision iris claw technique was hindered by data unavailability, highlighting critical methodological gaps in the existing literature, particularly the inconsistency in incision types and surgical techniques across studies. Given the limited number of non-randomised comparative studies and unequal group sizes, these findings should be interpreted cautiously and considered hypothesis-generating rather than definitive. Future studies should aim to standardize operative techniques and follow consistent reporting frameworks to enable more accurate and generalisable comparisons between these two IOL fixation strategies.

## Supplementary Information

Below is the link to the electronic supplementary material.

Supplementary Material 1.
