# Supplementary material for: Comparison of Carlevale and Artisan retropupillary iris-claw intraocular lens fixation for managing aphakia: a systematic review and meta-analysis
Source: Graefes Arch Clin Exp Ophthalmol. 2026 May 6;264(9):2693–702. doi: 10.1007/s00417-026-07263-8 (PMC13578012; doi:10.1007/s00417-026-07263-8)
Supplement: Supplementary file 1 [file 417_2026_7263_MOESM1_ESM.docx]

Figure S1: Study selection process

**Supplementary S1.** Search strategy

| Database | Search Strategy |
| --- | --- |
| PubMed | ("Aphakia"[Mesh] OR aphakic OR "Lenses, Intraocular"[Mesh] OR "Lens Implantation, Intraocular"[Mesh] OR "Lens Implant*" OR Lens OR "Cataract Extraction"[Mesh] OR "Lens Subluxation"[Mesh] OR "Lens Capsule, Crystalline"[Mesh]) **AND** (Carlevale OR FIL-SSF OR "FIL SSF" OR "Foldable Intraocular Lens" OR Sutureless OR "scleral-fixated" OR (Intrascleral AND fix) OR (scleral AND fix) OR (transscleral AND fix) OR "Intrascleral haptic fixation") **AND** (Artisan OR Iris-claw OR Retro-pupillary OR "Retropupillary" OR "Iris suture" OR (iris AND fix) OR Iris-clip) |
| Embase | ('aphakia'/exp OR aphakic OR 'lenses, intraocular'/exp OR 'lens implantation, intraocular'/exp OR 'lens implant*' OR lens OR 'cataract extraction'/exp OR 'lens subluxation'/exp OR 'lens capsule, crystalline'/exp) **AND** ('carlevale' OR 'FIL-SSF' OR 'FIL SSF' OR 'foldable intraocular lens' OR sutureless OR 'scleral-fixated' OR (intrascleral AND fix) OR (scleral AND fix) OR (transscleral AND fix) OR 'intrascleral haptic fixation') **AND** ('artisan' OR 'iris-claw' OR 'retropupillary' OR 'iris suture' OR (iris AND fix) OR 'iris-clip') |
| Cochrane | (aphakia OR aphakic OR "lenses, intraocular" OR "lens implantation, intraocular" OR "lens implant*" OR lens OR "cataract extraction" OR "lens subluxation" OR "lens capsule, crystalline") **AND** (Carlevale OR FIL-SSF OR "FIL SSF" OR "foldable intraocular lens" OR sutureless OR "scleral-fixated" OR (intrascleral AND fix) OR (scleral AND fix) OR (transscleral AND fix) OR "intrascleral haptic fixation") **AND** (Artisan OR Iris-claw OR retropupillary OR "iris suture" OR (iris AND fix) OR Iris-clip) |

**Supplementary S2. Methods of handling missing data**

In Aziria et al. [13], patients undergoing iris-claw implantation were separated depending on the type of incision, corneal versus sclerocorneal. The data of both were combined for the following outcomes: post-operative BCVA, post-operative astigmatism, mean operating time, using the following formulas:

$$Sample size: N_{1}+ N_{2}$$

$$Mean: \frac{N_{1} M_{1} + N_{2} M_{2}}{N_{1}+ N_{2}}$$

$$SD= \sqrt{\frac{(N_{1} - 1) S{D_{1}}^{2} + (N_{2} - 1) S{D_{2}}^{2} + \frac{N_{1} N_{2}}{N_{1}+ N_{2}}({M_{1}}^{2}+ {M_{2}}^{2} - 2M_{1}M_{2})}{N_{1}+ N_{2} - 1}}$$

In studies where the post-operative BCVA was not reported, post-operative BCVA was determined using the following formula, where p = 0.5 in the absence of study-specific correlation values:

$$Postop BCVA = Preop BCVA - Improvement in BCVA$$

$$SD = \sqrt{S{{D^{2}}_{pre}} + S{D^{2}}_{improvement} - 2 \cdot p \cdot SD_{pre}\cdot SD_{improvement}}$$

In studies where postoperative astigmatism was not reported, postoperative astigmatism was determined using the following formula, where p = 0.5 in the absence of study-specific correlation values:

$$Postop Astigmatism = Preop Astigmatism + SIA$$

$$SD = \sqrt{S{{D^{2}}_{preop}} + S{D^{2}}_{SIA} - 2 \cdot p \cdot SD_{preop}\cdot SD_{SIA}}$$

In studies where SIA was not reported, SIA was determined using the following formula, where p = 0.5 in the absence of study-specific correlation values:

$$SIA = Postop Astigmatism - Preop Astigmatism$$

$$SD= \sqrt{S{{D^{2}}_{postop}} + S{D^{2}}_{preop} - 2 \cdot p \cdot SD_{postop}\cdot SD_{preop}}$$

A sensitivity analysis was performed using correlation values of *p = 0.3* and *p = 0.7* to assess the robustness of our results to the choice of correlation coefficient in the imputation formulas. The results remained consistent, and the corresponding forest plots are provided below.

Forest plots of endpoints Post-operative BCVA, Post-operative astigmatism and Surgically induced astigmatism assessed using two correlation coefficients *(p = 0.3 and p = 0.7 respectively)* in the absence of specific data.

**Fig. S2.1** Postoperative BCVA (ρ = 0.3). No significant difference in outcome

**Fig. S2.2** Postoperative BCVA (ρ = 0.7). No significant difference in outcome

**Fig. S2.3** Postoperative astigmatism (ρ = 0.3). No significant difference in outcome

**Fig. S2.4** Postoperative astigmatism (ρ = 0.7). No significant difference in outcome

**Fig. S2.5** Surgically induced astigmatism (ρ = 0.3). No difference in outcome

**Fig. S2.6** Surgically induced astigmatism (ρ = 0.7). No difference in outcome.

**Supplementary S3. Table 1**

Table 1. Characteristics of Included Studies.

| **Study** | **Total Number of Eyes (C/R)** | **Age, y**† **(C/R)** | **Male, % (C/R)** | **Pre-op BCVA logMAR (C)*** | **Pre-op BCVA logMAR (R)*** | **Pre-op astigmatism (C)*** | **Pre-op astigmatism (R)*** | **Axial Length (C)*** | **Axial Length (R)*** | **Haptic Positioning from limbus (mm)** | **Follow up duration in months**† **(C/R)** |
| --- | --- | --- | --- | --- | --- | --- | --- | --- | --- | --- | --- |
| Obergassel 2024 | 51 (25/26) | 74.2/70.9 | 48/69.2 | 0.79 ± 0.51 | 0.63 ± 0.65 | 1.17 ± 1.18 | 1.32 ± 0.78 | N/A | N/A | 1.8 | 1.3 |
| Bontemps 2024 | 271 (96/175) | 70.3/70.5 | 70.8/62.9 | 0.70 ± 0.71 | 0.75 ± 0.72 | 1.45 ± 1.33 | 1.45 ± 1.58 | 24.2 ± 1.9 | 24.2 ± 1.8 | 2 | 9.3/11.5 |
| Aziria 2024 | 205 (63/142) | 69/70 | 70/64 | 0.75 ± 0.73 | 1.03 ± 0.87 | 1.20 ± 1.10 | 1.40 ± 1.60 | 23.9 ± 1.5 | 23.9 ± 2 | 2 | 2.53/7.72 |
| Bodin 2022 | 62 (25/37) | 65.9/64.5 | N/A | 0.70 ± 0.52 | 0.65 ± 0.51 | 1.90 ± 1.65 | 1.59 ± 4.16 | N/A | N/A | 1.5 | 3.3 |
| Seknazi 2021 | 42 (20/22) | 72.9/76.3 | 50/54.5 | NA | NA | NA | NA | 24.1 ± 1.5 | 23.8 ± 2.3 | 2 | 6.42/6.19 |

†: Mean; C: Carlevale; R: Retropupillary Iris-Claw; *(Mean ± SD); NA: Not available; BCVA: best corrected distance visual acuity; Astigmatism values are reported as absolute magnitudes (dioptres)

**Supplementary Figure S4. Fig. S4.1 to S4.12 captions**

**Fig. S4.1** Subgroup analysis of surgically induced astigmatism (SIA) comparing Carlevale and iris-claw IOLs by incision type. Carlevale showed significantly lower SIA in the corneal incision subgroup (MD −1.13 D; 95% CI −1.69 to −0.57; p < 0.01), but not in the mixed sclerocorneal subgroup. Significant heterogeneity was observed overall (I² = 73.7%), which resolved in the stratified analysis (p for subgroup difference = 0.0063)

**Fig. S4.2** Comparison of postoperative astigmatism between Carlevale and iris-claw IOLs. No statistically significant difference was observed (MD −0.23 D; 95% CI −0.53 to 0.07; p = 0.14), with no heterogeneity across studies (I² = 0%)

**Fig. S4.3** Forest plot comparing mean absolute refractive error (MARE) between Carlevale and iris-claw IOLs. No statistically significant difference was observed (MD −0.22 D; 95% CI −0.62 to 0.19; p = 0.30), substantial heterogeneity was present across studies (I² = 81.7%)

**Fig. S4.4** Leave-one-out sensitivity analysis for mean absolute refractive error (MARE). Exclusion of individual studies did not substantially reduce heterogeneity (I² range: 59.5% to 85.7%), indicating no single study accounted for the observed inconsistency

**Fig. S4.5** Subgroup analysis of MARE by incision type. The corneal incision subgroup showed significantly more favourable and consistent outcomes with Carlevale IOLs (MD −0.58 D; 95% CI −0.82 to −0.33; p < 0.01; I² = 0%)

**Fig. S4.6** Forest plot of Mean Refractive Error. Carlevale IOLs were associated with a significantly more myopic result compared to iris-claw lenses (MD −0.66 D; 95% CI −0.87 to −0.46; p < 0.01; I² = 31.3%)

**Fig. S4.7** Forest plot of operating time. Carlevale IOL implantation was associated with a significantly longer mean operating time (MD 11.89 minutes; 95% CI 5.20 to 18.58; p < 0.01; I² = 80.2%)

**Fig. S4.8** Leave-one-out analysis of operating time. Heterogeneity dropped to 0% upon exclusion of Bodin et al., identifying it as the primary source of variability. The refined pooled estimate after exclusion was 15.17 minutes (95% CI: 12.44 to 17.89; p < 0.01)

**Supplementary Figure S5.1.** Forest plots of post-operative complications reported in each group

**Fig. S5.1. IOL dislocation** Carlevale significantly reduced the odds of IOL dislocation (OR 0.16; 95% CI 0.03 to 0.87; p = 0.034; I² = 0%).

**Fig. S5.2 Cystoid Macular Oedema** No significant difference between groups (OR 0.91; 95% CI 0.45 to 1.83; p = 0.79; I² = 32.3%).

**Fig. S5.3 Hypotony** No significant difference observed (OR 0.95; 95% CI 0.18 to 5.07; p = 0.95; I² = 0%).

**Fig. S5.4 Retinal detachment** No significant difference between Carlevale and iris-claw IOLs (OR 1.33; 95% CI 0.32 to 5.51; p = 0.69; I² = 0%)

**Fig S5.5 Intravitreous Haemorrhage** No significant difference between groups (OR 1.41; 95% CI 0.47 to 4.24; p = 0.55; I² = 0%)

**Fig S5.6 Hyphaema** No statistically significant difference observed (OR 0.33; 95% CI 0.05 to 1.98; p = 0.23; I² = 0%)

**Supplementary Figure S6.** Risk of bias summary for non-randomized studies (ROBINS-I)

*ROBINS-I tool: Sterne et al. BMJ 2016;355:i4919.*

**Supplementary Figure S7.** Funnel plots of primary outcome Post-operative BCVA and Mean refractive error (MRE): The Egger’s test value for primary and secondary outcomes is above 0.05 and represents no significant publication bias

Post-operative BCVA

Mean Refractive Error (MRE)

**Supplementary Figure S8**. Leave-one-out analyses of outcomes

**Fig. S8.1** **Mean refractive error (MRE):** Exclusion of Obergassel et al. eliminated heterogeneity (I² = 0%) without affecting the pooled effect.

**Fig S8.2** **Postoperative astigmatism** No individual study significantly influenced the pooled estimate or heterogeneity.

**Supplementary Figure S9.** Stratifying outcomes by incision type

**Fig S9.1 Postoperative BCVA** No subgroup differences were detected (p = 0.45), with all incision types showing comparable visual outcomes between Carlevale and iris-claw IOLs

**Fig S9.2 Mean Refractive Error** Subgroup analysis by incision type showed consistently more myopic outcomes with Carlevale IOLs across all groups, with no significant subgroup differences (p = 0.09)

**Fig. S9.3 Mean operating time** Significant subgroup differences were observed (p = 0.0007). These differences are likely driven by study-level procedural factors (e.g. vitrectomy) rather than incision type alone

**Fig. S9.4 Postoperative astigmatism** No significant subgroup differences were found (p = 0.84), and all estimates showed comparable outcomes between Carlevale and iris-claw IOLs.

**Supplementary Figure S10**. Baujat plots of outcomes

**Fig S10.1 Postoperative BCVA** Aziria et al. (2024) contributed most to heterogeneity and had the strongest influence on the overall effect estimate

**Fig S10.2 Postoperative astigmatism** Bontemps et al. (2024) contributed most to heterogeneity and had the strongest influence on the overall effect estimate

**Fig S10.3 MARE** Obergassel et al. (2024) contributed most to heterogeneity and had the strongest influence on the overall effect estimate

**Fig S10.4 Mean Refractive Error** Obergassel et al. (2024) contributed most to heterogeneity and had the strongest influence on the overall effect estimate

**Fig S10.5 Operating time** Bodin et al. (2022) contributed most to heterogeneity and had the strongest influence on the overall effect estimate

**Fig S10.6 Surgically induced astigmatism** Obergassel et al. (2024) and Seknazi et al. (2021) jointly contributed most to heterogeneity and influenced the overall effect estimate

**Supplementary Figure S11.** GRADE Assessment

| **Carlevale IOL compared to Artisan IOL for Patients with aphakia or compromised capsular support requiring intraocular lens (IOL) implantation** | | | | | |
| --- | --- | --- | --- | --- | --- |
| **Patient or population:** Patients with aphakia or compromised capsular support requiring intraocular lens (IOL) implantation  **Setting:**  **Intervention:** Carlevale IOL  **Comparison:** Artisan IOL | | | | | |
| **Outcomes** | **№ of participants (studies) Follow-up** | **Certainty of the evidence (GRADE)** | **Relative effect (95% CI)** | **Anticipated absolute effects** | |
|  |  |  |  | **Risk with Artisan IOL** | **Risk difference with Carlevale IOL** |
| BCVA | 610 (5 non-randomised studies) | ⨁⨁◯◯ Low^a,b^ | - |  | MD **0.01 lower** (0.13 lower to 0.11 higher) |
| Post-op astigmatism | 589 (4 non-randomised studies) | ⨁⨁⨁◯ Moderate^a^ | - |  | MD **0.23 lower** (0.53 lower to 0.07 higher) |
| Surgically induced astigmatism | 631 (5 non-randomised studies) | ⨁⨁◯◯ Low^a,c^ | - |  | MD **0.53 lower** (1.03 lower to 0.04 lower) |
| Mean Refractive Error | 426 (4 non-randomised studies) | ⨁⨁◯◯ Low^a,d^ | - |  | MD **0.66 lower** (0.87 lower to 0.46 lower) |
| Mean Absolute Refractive Error | 426 (4 non-randomised studies) | ⨁◯◯◯ Very low^a,e,f^ | - |  | MD **0.22 lower** (0.62 lower to 0.19 higher) |
| Mean operating time | 568 (4 non-randomised studies) | ⨁⨁◯◯ Low^a,g^ | - |  | MD **11.89 higher** (5.2 higher to 18.58 higher) |
| ***The risk in the intervention group** (and its 95% confidence interval) is based on the assumed risk in the comparison group and the **relative effect** of the intervention (and its 95% CI).  **CI:** confidence interval; **MD:** mean difference; **OR:** odds ratio | | | | | |
| **GRADE Working Group grades of evidence** **High certainty:** we are very confident that the true effect lies close to that of the estimate of the effect. **Moderate certainty:** we are moderately confident in the effect estimate: the true effect is likely to be close to the estimate of the effect, but there is a possibility that it is substantially different. **Low certainty:** our confidence in the effect estimate is limited: the true effect may be substantially different from the estimate of the effect. **Very low certainty:** we have very little confidence in the effect estimate: the true effect is likely to be substantially different from the estimate of effect. | | | | | |

#### Explanations

a. Outcomes carried out by studies with moderate to high and/or serious risk of bias

b. High heterogeneity with I^2^ = 43%

c. High heterogeneity with I^2^ = 74%

d. Moderately high heterogeneity with I^2^ = 36%

e. High heterogeneity with I^2^ = 83%

f. 95% CI of MD includes 0, with p >0.05

g. High heterogeneity with I^2^ = 80%

h. Moderately high heterogeneity with I^2^ = 32%

i. 95% CI range includes odd ratio of 1
